# Clinical Outcomes of Different Generation EGFR TKIs in Susceptible EGFR‐Mutated Advanced Nonsmall‐Cell Lung Cancer

**DOI:** 10.1002/kjm2.70105

**Published:** 2025-09-25

**Authors:** Chia‐Yu Kuo, Tien‐Chi Huang, Chih‐Jen Yang, Mei‐Hsuan Lee, Jui‐Ying Lee, Ying‐Ming Tsai, Kuan‐Li Wu, Cheng‐Hao Chuang, Inn‐Wen Chong, Jen‐Yu Hung

**Affiliations:** ^1^ Division of Pulmonary and Critical Care Medicine, Department of Internal Medicine Kaohsiung Medical University Hospital, Kaohsiung Medical University Kaohsiung Taiwan; ^2^ Division of Pulmonary and Critical Care Medicine, Department of Internal Medicine Kaohsiung Municipal Siaogang Hospital Kaohsiung City Taiwan; ^3^ Division of Cardiology, Department of Internal Medicine Kaohsiung Medical University Hospital, Kaohsiung Medical University Kaohsiung City Taiwan; ^4^ School of Medicine, College of Medicine Kaohsiung Medical University Kaohsiung Taiwan; ^5^ School of Post‐Baccalaureate Medicine, College of Medicine Kaohsiung Medical University Kaohsiung Taiwan; ^6^ Division of Chest Surgery, Department of Surgery Kaohsiung Medical University Hospital, Kaohsiung Medical University Kaohsiung Taiwan

**Keywords:** epidermal growth factor receptor, lung cancer, tyrosine kinase inhibitor

## Abstract

Epidermal growth factor receptor (EGFR) tyrosine kinase inhibitors (TKIs) are indicated for advanced lung adenocarcinoma patients harboring susceptible EGFR mutations. The aim of this retrospective study was to compare the effectiveness of different generations of EGFR TKIs. We enrolled 421 patients with stage IV lung adenocarcinoma and sensitizing EGFR mutations receiving an EGFR‐TKI as their first‐line therapy, including first‐generation (1st G, gefitinib and erlotinib), second‐generation (2nd G, afatinib), and third‐generation (3rd G, osimertinib) EGFR TKIs. The median progression free survival (PFS) (12.10 vs. 16.67 months vs. not reached; *p* = 0.0002) and overall survival (OS) (31.23 vs. 45.97 months vs. not reached; *p* = 0.0215) were significantly different between different generations of EGFR TKIs. 3rd G EGFR TKI provided the best PFS, particularly in patients with exon 19 deletion. The patients receiving 1st G EGFR TKIs (*p* = 0.005), with exon 19 deletion (*p* = 0.001) and PFS ≥ 270 days (*p* = 0.012) had a significantly higher T790M mutation rate. There was no survival difference between the patients receiving frontline 3rd G EGFR TKI and those receiving 3rd G EGFR TKI as sequential therapy (median OS 46.60 months vs. not reached, *p* = 0.1941). The OS of the patients who did not receive 3rd G EGFR TKI as sequential therapy was significantly worse than those receiving 3rd G EGFR TKI as first‐line therapy (median OS 22.47 months vs. not reached, *p* = 0.0042). In conclusion, 3rd G EGFR TKI may provide better survival benefits as first‐line therapy for patients harboring sensitizing EGFR mutations, particularly those with exon 19 deletion.

## 1 Introduction

Nonsmall cell lung cancer (NSCLC) is the leading cause of cancer‐related mortality worldwide, and the most common cell type is adenocarcinoma [1]. The standard treatment for NSCLC patients was traditional chemotherapy [2]; however, since the development of epidermal growth factor receptor (EGFR) tyrosine kinase inhibitors (TKIs), clinical practice has changed. The incidence of EGFR mutations in NSCLC patients is approximately 40%–60% in Asia [3, 4], compared to only 10%–20% in Western countries [5]. In recent years, several phase III randomized trials have demonstrated that patients with NSCLC harboring EGFR mutations such as exon 21 L858R point mutation and exon 19 deletion have better clinical outcomes when treated with gefitinib, erlotinib, and afatinib compared to traditional chemotherapy [6, 7, 8, 9, 10]. In the past decade, EGFR TKIs have become established as the standard‐of‐care first‐line therapies for patients with NSCLC harboring EGFR mutations [11].

Despite a good initial response, acquired resistance almost always develops. The main resistance mechanism of first‐generation (1st G) and second‐generation (2nd G) EGFR TKIs is the development of the T790M mutation [12]. The T790M mutation rate after disease progression with a first‐line EGFR TKI is approximately 50% [13, 14]. Previous studies have reported that patients with the T790M mutation after EGFR TKI failure tend to have a better prognosis than those without the mutation [15, 16]. Osimertinib is a third‐generation (3rd G) EGFR TKI targeting EGFR sensitizing mutations, and it was proved to be effective in NSCLC patients with the T790M mutation after EGFR TKI failure [17]. Sequential therapy with osimertinib subsequent to gefitinib, erlotinib, and afatinib may allow for a longer chemotherapy‐free interval.

Recent studies have also shown the superior efficacy of osimertinib compared to 1st G EGFR TKIs in the first‐line therapy of EGFR mutation‐positive advanced NSCLC [18, 19]. We conducted the real‐world study to analyze the clinical outcomes of patients with NSCLC harboring sensitizing EGFR mutations receiving 1st G, 2nd G, and 3rd G EGFR TKIs as first‐line therapy. We also evaluated the clinical outcomes of 3rd G EGFR TKI as first‐line therapy or sequential therapy after 1st/2nd G EGFR TKIs in metastatic NSCLC patients harboring sensitizing EGFR mutations.

## 2 Patients and Methods

### 2.1 Patient Identification

**FIGURE 1:**
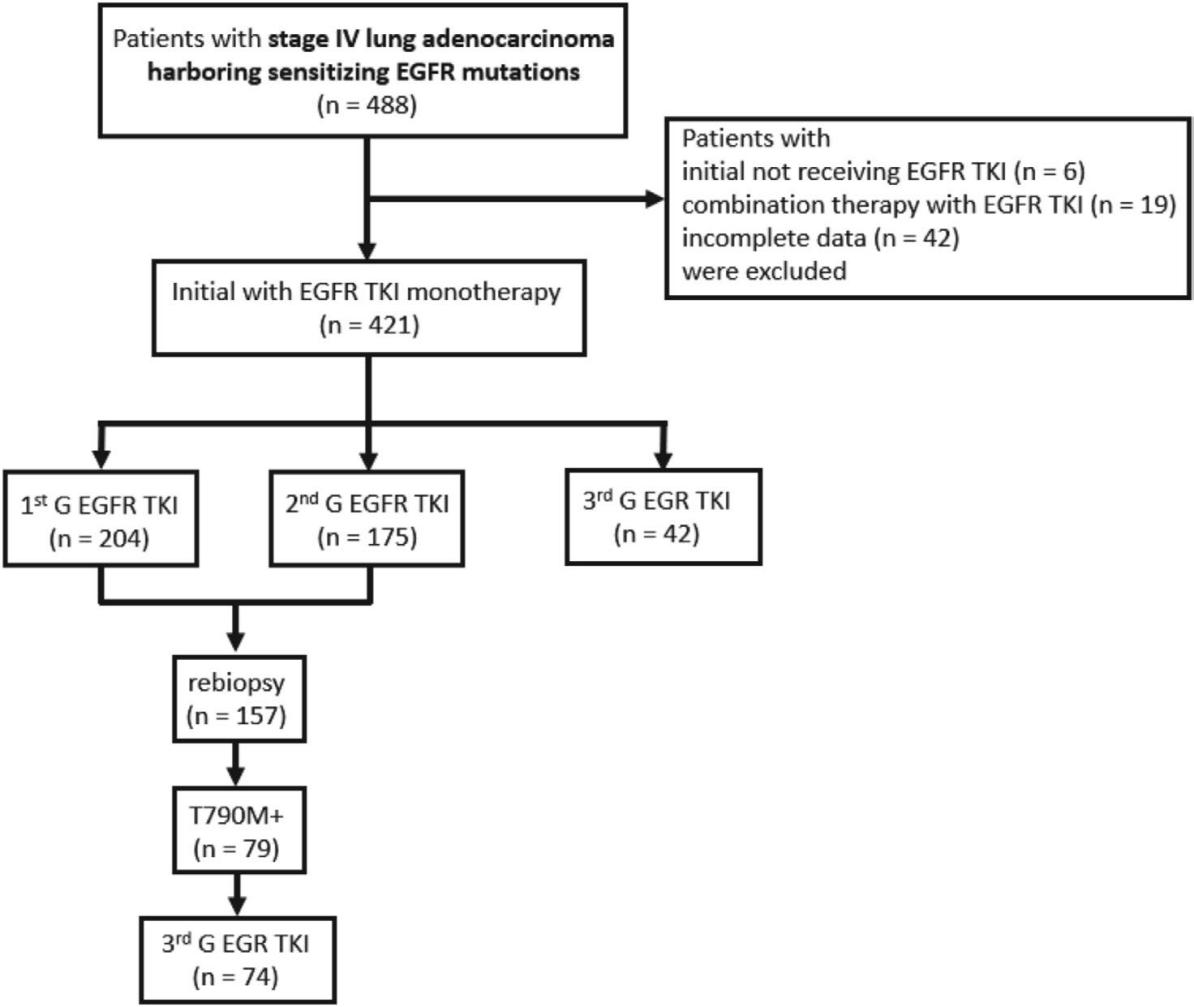
Flowchart of the study population. EGFR, epidermal growth factor receptor; TKI, tyrosine kinase inhibitor.

We enrolled patients with lung adenocarcinoma diagnosed and treated at three Kaohsiung Medical University (KMU)‐affiliated hospitals, including Kaohsiung Medical University Hospital (KMUH), Kaohsiung Municipal Ta‐Tung Hospital, and Kaohsiung Municipal Siaogang Hospital (Figure 1). The diagnosis of lung adenocarcinoma was confirmed pathologically according to the World Health Organization pathology classification, and cancer staging was confirmed by lung cancer teams according to the eighth version of the American Joint Committee on Cancer staging system. Genomic DNA extracted from tissue blocks was subjected to genotyping of exons 18–21 of the EGFR gene using real‐time polymerase chain reaction (PCR) (cobas EGFR Mutation Test v2, Roche Molecular Systems Inc., Pleasanton, CA). This ready‐to‐use kit can detect 42 somatic mutations in the EGFR gene, and we used a cobas z480 analyzer and a cobas 4800 analyzer (Roche) following the techniques reported in our previous studies [20, 21, 22, 23, 24, 25]. The Institutional Review Board (IRB) of KMUH approved this study (KMUHIRB‐G(II)‐20220027, KMUHIRB‐E(I)‐20250196) and waived the need for written informed consent from the patients.

We enrolled all patients diagnosed as stage IV lung adenocarcinoma with EGFR mutations between June 2018 and December 2021. Patients diagnosed with adenosquamous carcinoma were excluded from the study. All of the patients were naïve to systemic treatment and were treated with an EGFR TKI as their first‐line therapy, including 1st G (gefitinib and erlotinib), 2nd G (afatinib), and 3rd G (osimertinib) EGFR TKIs. Patients with initial therapy other than EGFR TKIs, with combination therapy (antiangiogenic agents or chemotherapeutic agents) with EGFR TKIs, and with incomplete data were excluded. Baseline clinical characteristics were recorded through retrospective review of medical records. Age at diagnosis, sex, Eastern Cooperative Oncology Group (ECOG) performance status (PS), EGFR mutations, site of metastasis, and the generation of EGFR TKI were included. No patients received radiation therapy for the main lesion prior to disease progression.

The initial treatment response was classified based on serial imaging studies using the revised Response Evaluation Criteria in Solid Tumors (RECIST 1.1) criteria. Progression‐free survival (PFS) and overall survival (OS) were defined as the time from the initiation of the EGFR TKI to the date of disease progression on an imaging examination and the date of death or August 2023, respectively.

### 2.2 Statistical Analysis

Categorical variables and continuous variables were compared using the Chi‐squared test and the independent samples *t* test, respectively. Survival times were estimated using the Kaplan–Meier method, and differences between groups were compared with the log‐rank test. Both univariable and multivariable Cox regression analyses were used to determine the predictive factors for PFS and OS, and hazard ratios (HR) with 95% confidence intervals (CIs) are presented. All statistical analyses were performed with STATA software (version 18 for Windows, StataCorp LLC). Statistical significance was set at a two‐tailed *p* < 0.05.

## 3 Results

We identified 488 patients with stage IV lung adenocarcinoma harboring sensitizing EGFR mutations. In the 488 patients, six patients with initial therapy other than EGFR TKIs, 19 patients receiving combination therapy with EGFR TKIs, and 42 patients with incomplete data were excluded. Four hundred and twenty‐one patients treated with EGFR TKIs as their first‐line systemic treatment were enrolled in our study (Table 1). Of the 421 patients, 204 received 1st G EGFR TKI, 175 received 2nd G EGFR TKI, and 42 received 3rd G EGFR TKI as first‐line therapy. Exon 19 deletion and exon 21 L858R were detected in the tumors of 194 (46%) and 195 (46%) patients, respectively. Rare mutations such as G719X, L861Q, and S768I were detected in the tumors of 31 (7%) patients. One of the 31 patients had de novo T790M. The median follow‐up time was 22.6 months. Smoking status and PS were similar among the three groups. However, sex (*p* = 0.027), age (*p* < 0.001), EGFR mutation type (*p* < 0.001), brain metastasis (*p* < 0.001), pleural metastasis/effusion (*p* = 0.010), and adrenal gland metastasis (*p* = 0.048) were significantly different among the three groups. There was no significant difference in median follow‐up duration (19.4, 23.9, 23.7 months, *p* = 0.113), disease control rate (DCR) (93%, 94%, 95%, *p* = 0.729) or ORR (74%, 74%, 81%, *p* = 0.606) among the three groups.

**TABLE 1:** Baseline characteristics of the study cohort.

Variables	All patients	1st G TKI	2nd G TKI	3rd G TKI	*p*
*n*	421	204	175	42	
Sex					0.027
Female	282 (67%)	149 (73%)	105 (60%)	28 (67%)	
Male	139 (33%)	55 (27%)	70 (40%)	14 (33%)	
Age (years)	67.6 ± 10.9	70.0 ± 10.8	65.2 ± 10.4	66.2 ± 11.7	< 0.001
< 65	159 (38%)	58 (28%)	82 (47%)	19 (45%)	0.001
≥ 65	262 (62%)	146 (72%)	93 (53%)	23 (55%)	
Smoking status					0.204
Current smoker	22 (5%)	9 (4%)	12 (7%)	1 (2%)	
Ex‐smoker	46 (11%)	18 (9%)	25 (14%)	3 (7%)	
Never smoker	353 (84%)	177 (87%)	138 (79%)	38 (90%)	
ECOG performance status					0.109
0–1	359 (85%)	172 (84%)	155 (89%)	32 (76%)	
≥ 2	62 (15%)	32 (16%)	20 (11%)	10 (24%)	
EGFR mutation					< 0.001
Exon 19 deletion	194 (46%)	89 (44%)	72 (41%)	33 (79%)	
Exon 21 L858R	195 (46%)	113 (55%)	73 (42%)	9 (21%)	
19 deletion + L858R	1 (1%)	0 (0%)	1 (1%)	0 (0%)	
Others	31 (7%)	2 (1%)	29 (16%)	0 (0%)	
Metastasis site					
Brain	143 (34%)	92 (45%)	41 (23%)	10 (24%)	< 0.001
Lung	246 (58%)	113 (55%)	110 (63%)	23 (55%)	0.298
Pleura/pleural effusion	190 (45%)	95 (47%)	68 (39%)	27 (64%)	0.010
Pericardial metastasis/effusion	49 (12%)	30 (15%)	15 (9%)	4 (10%)	0.161
Bone	209 (50%)	100 (49%)	90 (51%)	19 (45%)	0.748
Liver	55 (13%)	29 (14%)	21 (12%)	5 (12%)	0.794
Adrenal gland	46 (11%)	30 (15%)	12 (7%)	4 (10%)	0.048
Other	37 (9%)	17 (8%)	15 (9%)	5 (12%)	0.751
Median follow‐up time (month)	22.6	19.4	23.9	23.7	0.113
Initial treatment response					
CR	3 (1%)	2 (1%)	1 (1%)	0 (0%)	
PR	311 (74%)	149 (73%)	128 (73%)	34 (81%)	
SD	80 (19%)	38 (19%)	36 (20%)	6 (14%)	
PD	27 (6%)	15 (7%)	10 (6%)	2 (5%)	
Disease control rate	394 (94%)	189 (93%)	165 (94%)	40 (95%)	0.729
Objective response rate	314 (75%)	151 (74%)	129 (74%)	34 (81%)	0.606
PD before data cutoff date	293 (70%)	152 (75%)	123 (70%)	18 (43%)	0.006
Subsequent therapy status after PD					
Subsequent therapy	206 (70%)	95 (63%)	95 (77%)	16 (89%)	
No subsequent therapy	87 (30%)	57 (37%)	28 (23%)	2 (11%)	

*Note*: Data are presented in *n* (%) or median (interquartile range). *p* values were assessed with Fisher's exact test or Wilcoxon rank‐sum test.

Abbreviations: 1st G, first‐generation; 2nd G, second‐generation; 3rd G, third‐generation; CR, complete response; ECOG, Eastern Cooperative Oncology Group; EGFR, epidermal growth factor receptor; PD, progressive disease; PR, partial response; SD, stable disease; TKI, tyrosine kinase inhibitor.

**FIGURE 2:**
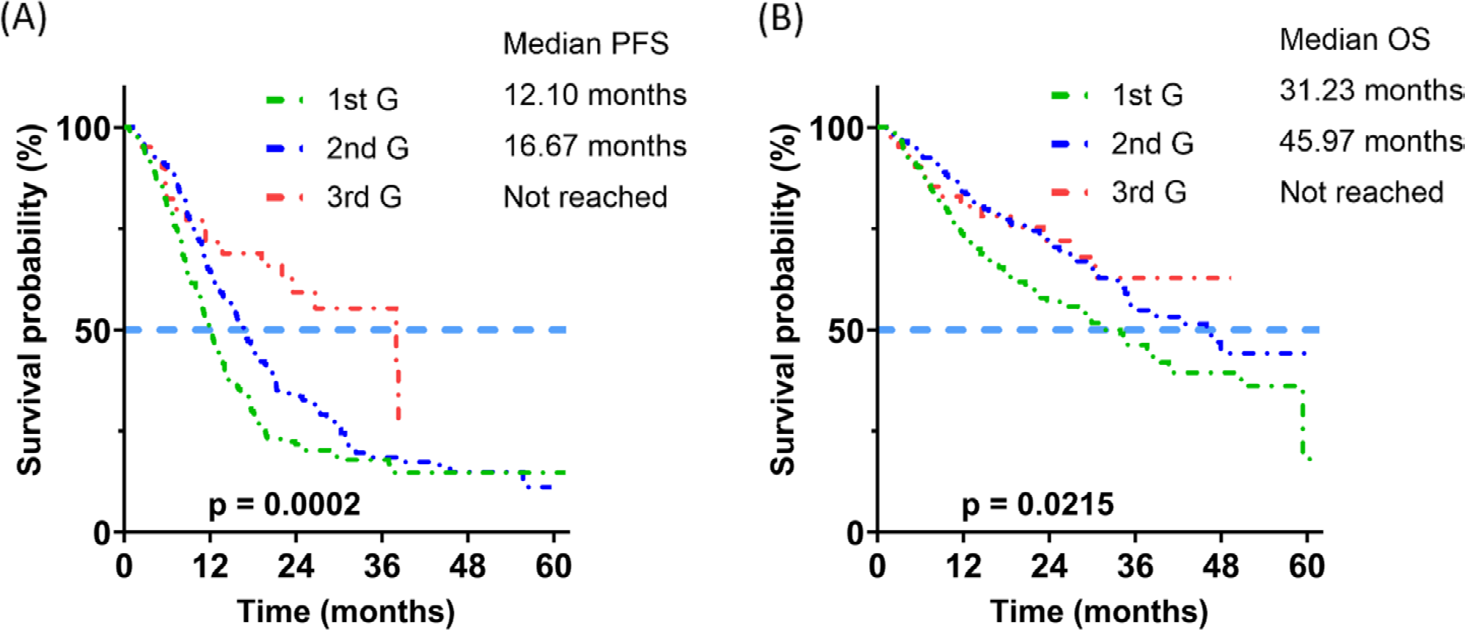
Progression‐free survival (PFS) (A) and overall survival (OS) (B) of the patients who received different generations of epidermal growth factor receptor tyrosine kinase inhibitors.

**FIGURE 3:**
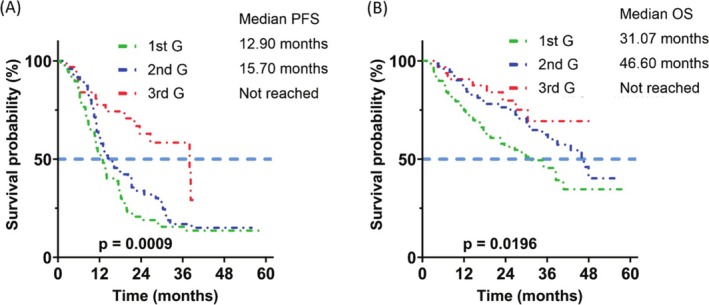
Progression‐free survival (PFS) (A) and overall survival (OS) (B) of the patients who received different generations of epidermal growth factor receptor tyrosine kinase inhibitors with exon 19 deletion.

**FIGURE 4:**
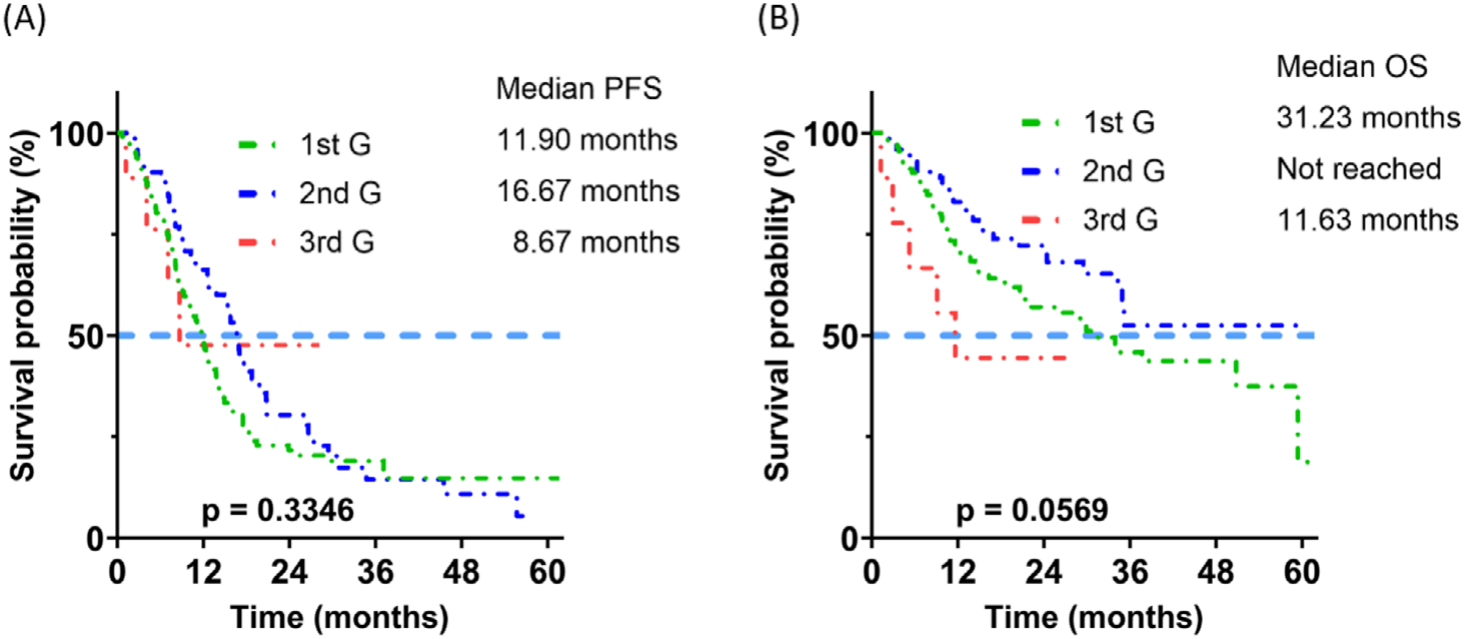
Progression‐free survival (PFS) (A) and overall survival (OS) (B) of the patients who received different generations of epidermal growth factor receptor tyrosine kinase inhibitors with L858R mutation.

**FIGURE A1:**
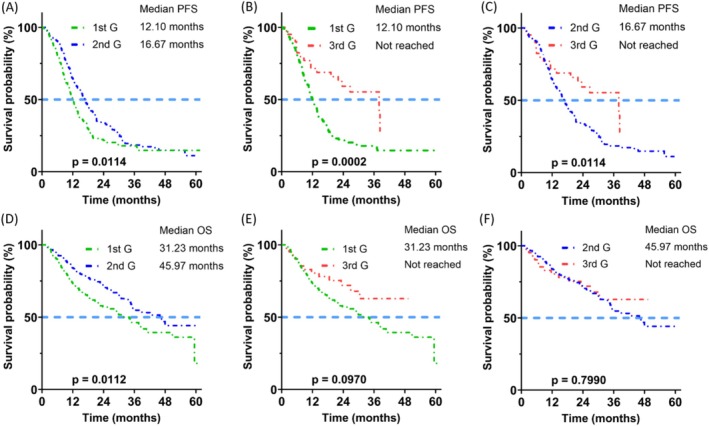
Progression‐free survival (PFS) (A, B, C) and overall survival (OS) (D, E, F) of the patients who received 1st G versus 2nd G (A, D), 2nd G versus 3rd G (B, E), and 1st G versus 3rd G (C, F) epidermal growth factor receptor tyrosine kinase inhibitors.

**FIGURE A2:**
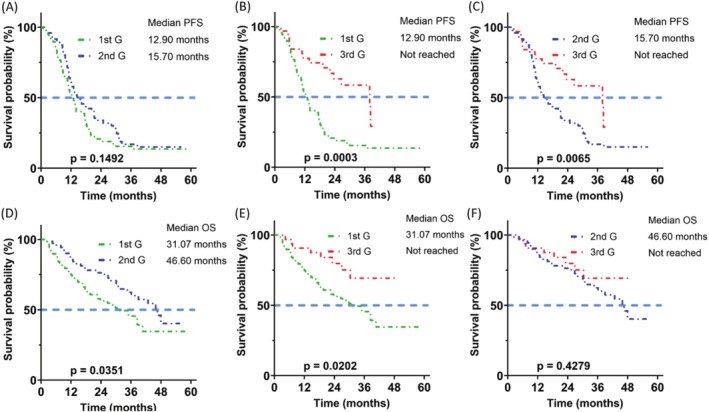
Progression‐free survival (PFS) (A, B, C) and overall survival (OS) (D, E, F) of the patients with exon 19 deletions who received 1st G versus 2nd G (A, D), 2nd G versus 3rd G (B, E), and 1st G versus 3rd G (C, F) epidermal growth factor receptor tyrosine kinase inhibitors.

**FIGURE A3:**
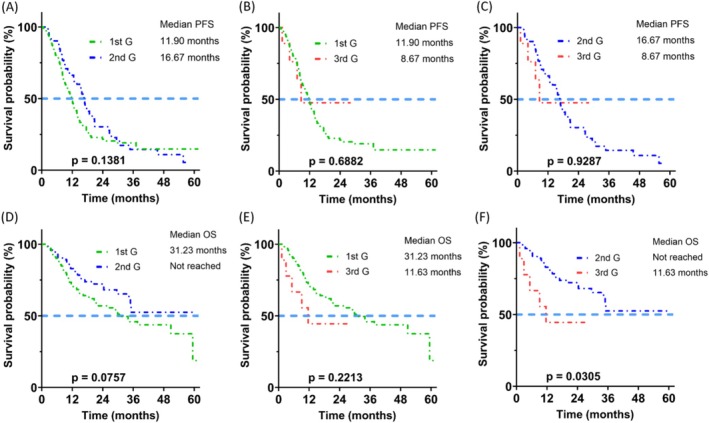
Progression‐free survival (PFS) (A, B, C) and overall survival (OS) (D, E, F) of the patients with L858R who received 1st G versus 2nd G (A, D), 2nd G versus 3rd G (B, E), and 1st G versus 3rd G (C, F) epidermal growth factor receptor tyrosine kinase inhibitors.

The PFS was different between the patients receiving 1st G, 2nd G, and 3rd G EGFR TKIs as first‐line therapy (median 12.10, 16.67 months, or not reached, respectively; *p* = 0.0002) (Figure 2A). 3rd G EGFR TKIs were associated with longer PFS than 1st G (*p* = 0.0002) and 2nd G (*p* = 0.0114) EGFR TKIs (Figure A1A, A1B, A1C). The OS was also different between the three groups (median 31.23, 45.97 months, or not reached, respectively; *p* = 0.0215) (Figure 2B). The patients receiving 2nd G EGFR TKI had a significantly better OS than those who received 1st G EGFR TKIs (*p* = 0.0112) (Figure A1D). In the subgroup of patients with exon 19 deletion, the PFS and OS were significantly different between the three groups (PFS: median 12.9, 15.70 months, or not reached, respectively; *p* = 0.0009) (Figure 3A); and OS (median 31.07, 46.60 months, or not reached, respectively; *p* = 0.0196) (Figure 3B). The patients with exon 19 deletion who received the 3rd G EGFR TKI had a better PFS than those who received 1st G (*p* = 0.0003) and 2nd G (*p* = 0.0065) EGFR TKIs (Figure A2A, A2B, A2C). The patients who received 2nd G (*p* = 0.0351) and 3rd G (*p* = 0.0202) EGFR TKIs had a significantly better OS (Figure A2D, A2E) than the patients who received 1st G EGFR TKI. In the subgroup of patients with exon 21 L858R mutation, the PFS (median 11.90, 16.67, or 8.67 months, respectively; *p* = 0.3346) and OS (median 31.23 months, not reached, or 11.63 months, respectively; *p* = 0.0569) were similar between the three groups (Figure 4). There was no significant difference in PFS between the patients who received 1st G versus 2nd G (*p* = 0.1381), 1st G versus 3rd G (*p* = 0.6882), and 2nd G versus 3rd G (*p* = 0.9287) EGFR TKIs (Figure A3A, A3B, A3C). The OS was similar between the patients with the exon 21 L858R mutation who received 1st G versus 2nd G (*p* = 0.0757) and 1st G versus 3rd G (*p* = 0.2213) EGFR TKIs (Figure A3D, A3E). The patients with exon 21 L858R mutation who received 2nd G EGFR TKI had a better OS than those who received 3rd G EGFR TKI (*p* = 0.0305) (Figure A3F).

Cox regression analysis was used to identify the risk factors associated with PFS and OS. Multivariable analysis with backward variable selection confirmed that first‐line therapy with 3rd G EGFR TKI (HR [95% CI] = 0.38 [0.23–0.64], *p* < 0.001) was an independent prognostic factor of better PFS, while pleural metastasis/effusion (HR [95% CI] = 1.32 [1.03–1.71], *p* = 0.029) and bone metastasis (HR [95% CI] = 1.93 [1.49–2.50], *p* < 0.001) were independent prognostic factors of poorer PFS (Table 2). Multivariate analysis with backward variable selection showed that independent prognostic factors of poorer OS included age ≥ 65 years (HR [95% CI] = 1.47 [1.05–2.07], *p* = 0.024), ECOG PS ≥ 2 (HR [95% CI] = 1.70 [1.14–2.54], *p* = 0.010), lung metastasis (HR [95% CI] = 1.40 [1.01–1.93], *p* = 0.044), bone metastasis (HR [95% CI] = 1.55 [1.12–2.15], *p* = 0.009) and liver metastasis (HR [95% CI] = 1.60 [1.07–2.39], *p* = 0.022) (Table 3).

**TABLE 2:** Cox regression analysis to identify factors associated with progression free survival.

Variables	Univariate		Multivariable model	
	HR [95% CI]	*p*	HR [95% CI]	*p*
EGFR TKI: (vs. 1st G TKI)				
2nd G TKI	0.74 [0.58–0.94]	0.013	0.79 [0.61–1.03]	0.084
3rd G TKI	0.40 [0.24–0.65]	< 0.001	0.38 [0.23–0.64]	< 0.001
Male (vs. female)	1.11 [0.87–1.42]	0.400	1.09 [0.81–1.46]	0.569
Age (≥ 65 vs. < 65)	0.89 [0.71–1.13]	0.340	0.86 [0.66–1.11]	0.256
ECOG PS (≥ 2 vs. ≤ 1)	1.29 [0.93–1.79]	0.123	1.17 [0.82–1.67]	0.373
Smoking status (vs. never smoker)				
Current smoker	1.06 [0.65–1.73]	0.819	0.89 [0.52–1.51]	0.657
EX‐smoker	1.13 [0.78–1.64]	0.508	1.19 [0.76–1.85]	0.443
Mutation type: (vs. Exon 19 deletion)				
L858R	1.26 [0.99–1.59]	0.058	1.16 [0.91–1.48]	0.234
Others	0.83 [0.51–1.34]	0.447	0.68 [0.40–1.14]	0.144
19 deletion + L858R		1.000		1.000
Brain metastasis (vs. no)	1.38 [1.08–1.76]	0.009	1.21 [0.91–1.59]	0.188
Lung metastasis (vs. no)	1.14 [0.90–1.44]	0.265	1.03 [0.81–1.33]	0.789
Pleural metastasis/effusion (vs. no)	1.18 [0.93–1.48]	0.168	1.32 [1.03–1.71]	0.029
Bone metastasis (vs. no)	1.86 [1.47–2.36]	< 0.001	1.93 [1.49–2.50]	< 0.001
Liver metastasis (vs. no)	1.58 [1.14–2.19]	0.006	1.20 [0.85–1.70]	0.296
Pericardial metastasis/effusion (vs. no)	1.24 [0.87–1.78]	0.234	0.83 [0.55–1.25]	0.362
Adrenal metastasis (vs. no)	1.23 [0.84–1.78]	0.285	1.26 [0.84–1.89]	0.264
Other metastasis (vs. no)	0.93 [0.60–1.42]	0.723	0.94 [0.60–1.47]	0.777

*Note*: Data are presented in hazard ratio (HR) [95% confidence interval].

Abbreviations: 1st G, first‐generation; 2nd G, second‐generation; 3rd G, third‐generation; ECOG, Eastern Cooperative Oncology Group; EGFR, epidermal growth factor receptor; PS, performance status; TKI, tyrosine kinase inhibitor.

**TABLE 3:** Cox regression analysis to identify factors associated with overall survival.

Variables	Univariate		Multivariable model	
	HR [95% CI]	*p*	HR [95% CI]	*p*
EGFR TKI: (vs. 1st G TKI)				
2nd G TKI	0.67 [0.49–0.92]	0.012	0.74 [0.52–1.04]	0.086
3rd G TKI	0.62 [0.35–1.10]	0.103	0.65 [0.35–1.19]	0.165
Male (vs. female)	1.46 [1.08–1.97]	0.014	1.42 [0.98–2.06]	0.064
Age (≥ 65 vs. < 65)	1.49 [1.08–2.03]	0.014	1.47 [1.05–2.07]	0.024
ECOG PS (≥ 2 vs. ≤ 1)	1.90 [1.31–2.77]	0.001	1.70 [1.14–2.54]	0.010
Smoking status (vs. never smoker)				
Current smoker	1.48 [0.84–2.61]	0.177	1.29 [0.71–2.36]	0.406
EX‐smoker	1.59 [1.04–2.43]	0.033	1.30 [0.78–2.18]	0.315
Mutation type: (vs. Exon 19 deletion)				
L858R	1.14 [0.84–1.55]	0.389	1.02 [0.75–1.40]	0.879
Others	1.01 [0.55–1.87]	0.962	1.30 [0.78–2.18]	0.315
19 deletion + L858R		1.000		1.000
Brain metastasis (vs. no)	1.54 [1.14–2.09]	0.005	1.18 [0.83–1.68]	0.356
Lung metastasis (vs. no)	1.38 [1.01–1.87]	0.041	1.40 [1.01–1.93]	0.044
Pleural metastasis/effusion (vs. no)	1.20 [0.89–1.60]	0.229	1.35 [0.97–1.88]	0.079
Bone metastasis (vs. no)	1.75 [1.30–2.36]	< 0.001	1.55 [1.12–2.15]	0.009
Liver metastasis (vs. no)	1.93 [1.34–2.79]	< 0.001	1.60 [1.07–2.39]	0.022
Pericardial metastasis/effusion (vs. no)	1.55 [1.03–2.34]	0.036	1.03 [0.63–1.69]	0.908
Adrenal metastasis (vs. no)	1.82 [1.18–2.80]	0.007	1.60 [1.00–2.55]	0.050
Other metastasis (vs. no)	1.21 [0.73–2.03]	0.462	1.21 [0.70–2.07]	0.500

*Note*: Data are presented in hazard ratio (HR) [95% confidence interval].

Abbreviations: 1st G, first‐generation; 2nd G, second‐generation; 3rd G, third‐generation; ECOG, Eastern Cooperative Oncology Group; EGFR, epidermal growth factor receptor; PS, performance status; TKI, tyrosine kinase inhibitor.

Of the patients who received 1st G or 2nd G EGFR TKI as first‐line therapy, 275 had disease progression during the study period. Among these 275 patients, 157 received re‐biopsy for the T790M mutation, including 138 (88%) who underwent a tissue biopsy for formalin‐fixed and paraffin‐embedded tumor tissue samples, and 19 (12%) who underwent a liquid biopsy for circulating tumor DNA. Among the patients who received re‐biopsy, 79 (50%) patients were found T790M positive. The patients who received 1st G EGFR TKIs (*p* = 0.005), with exon 19 deletion (*p* = 0.001) and PFS ≥ 270 days (*p* = 0.012) had a significantly higher T790M mutation rate (Table 4). Of the 79 patients with T790M mutation, 74 received 3rd G EGFR TKI as sequential therapy. Of these 74 patients, 46 received 1st G EGFR TKI, while 28 received 2nd G EGFR TKI as their first‐line therapy, respectively. There were no significant differences in sex, smoking status, ECOG PS, or sites of metastasis between the patients who received 3rd G EGFR TKI as first‐line therapy or sequential therapy (Table 5).

**TABLE 4:** Baseline characteristics of the patients receiving re‐biopsy.

Variables	Number of patients	T790M (%)	*p*
Sex			0.447
Female	113	59 (52%)	
Male	44	20 (45%)	
Age (year)			0.453
< 65	75	41 (55%)	
≥ 65	82	38 (46%)	
Smoking status			0.482
Current smoker	4	1 (25%)	
EX‐smoker	14	6 (43%)	
Never smoker	139	72 (52%)	
ECOG performance status			0.593
0–1	143	71 (50%)	
≥ 2	14	8 (57%)	
TKI type			0.005
1st G	77	50 (65%)	
2nd G	80	29 (36%)	
EGFR mutation			0.001
Exon 19 deletion	75	48 (64%)	
Exon 21 L858R	76	31 (41%)	
Others	6	0 (0%)	
Biopsy type			0.784
Tissue biopsy	138	70 (51%)	
Liquid biopsy	19	9 (47%)	
PFS			0.012
PFS ≥ 270 days	111	63 (57%)	
PFS < 270 days	46	16 (35%)	

Abbreviations: 1st G, first‐generation; 2nd G, second‐generation; ECOG, Eastern Cooperative Oncology Group; EGFR, epidermal growth factor receptor; PFS, progression‐free survival; TKI, tyrosine kinase inhibitor.

**TABLE 5:** Baseline characteristics of the patients receiving 3rd G EGFR TKI as first‐line therapy and as sequential therapy after 1st G and 2nd G EGFR‐TKIs.

Variables	1st G/2nd G → 3rd G	3rd G first‐line	*p*
*n*	74	42	
Sex			0.380
Female	55 (74%)	28 (67%)	
Male	10 (26%)	14 (33%)	
Age (year)	64.6 ± 10.6	66.2 ± 11.7	0.471
< 65	37 (50%)	19 (45%)	
≥ 65	37 (50%)	23 (55%)	
Smoking status			0.916
Current smoker	1 (1%)	1 (2%)	
EX‐smoker	5 (7%)	3 (7%)	
Never smoker	68 (92%)	38 (90%)	
ECOG performance status			0.063
0–1	66 (89%)	32 (76%)	
≥ 2	8 (11%)	10 (24%)	
EGFR mutation			0.019
Exon 19 deletion	45 (61%)	33 (79%)	
Exon 21 L858R	29 (39%)	9 (21%)	
First‐line TKI			
1st G	46 (62%)	0	
2nd G	28 (38%)	0	
3rd G	0	42 (100%)	
Metastasis site			
Brain	30 (41%)	10 (24%)	0.068
Lung	46 (62%)	23 (55%)	0.435
Pleura/pleural effusion	34 (50%)	27 (64%)	0.137
Pericardial metastasis/effusion	13 (18%)	4 (10%)	0.239
Bone	44 (59%)	19 (45%)	0.139
Liver	13 (18%)	5 (12%)	0.418
Adrenal gland	3 (4%)	4 (10%)	0.234
Other	5 (7%)	5 (12%)	0.342

Abbreviations: 1st G, first‐generation; 2nd G, second‐generation; 3rd G, third‐generation; ECOG, Eastern Cooperative Oncology Group; EGFR, epidermal growth factor receptor; TKI, tyrosine kinase inhibitor.

**FIGURE 5:**
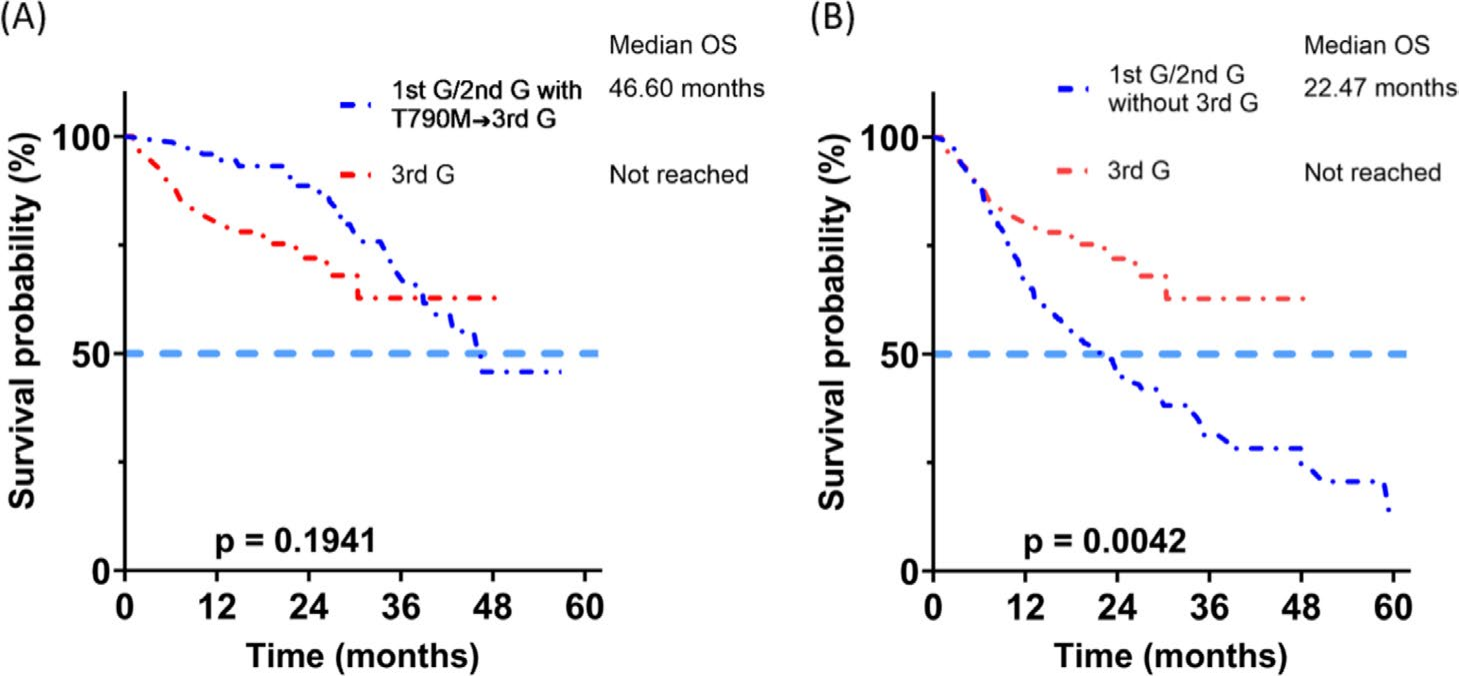
Overall survival (OS) of the patients who received third‐generation (3rd G) epidermal growth factor receptor (EGFR) tyrosine kinase inhibitor (TKI) as first‐line therapy versus sequential 3rd G EGFR TKI (A) and versus no 3rd G EGFR TKI sequential therapy (B) after 1st G or 2nd G EGFR TKIs.

The OS of the patients who received 3rd G EGFR TKI as sequential therapy was similar to those who received 3rd G EGFR TKI as first‐line therapy (median 46.60 months vs. not reached, *p* = 0.1941) (Figure 5A). Multivariate analysis with backward variable selection showed that the independent prognostic factors of poorer OS were male sex (HR [95% CI] = 2.71 [1.05–7.02], *p* = 0.039), lung metastasis (HR [95% CI] = 3.19 [1.38–7.41], *p* = 0.007), liver metastasis (HR [95% CI] = 3.75 [1.54–9.11], *p* = 0.004) and adrenal metastasis (HR [95% CI] = 5.20 [1.48–18.29], *p* = 0.010) (Table 6). Of the 275 patients with disease progression after treatment with 1st G or 2nd G EGFR TKIs, 173 did not receive 3rd G EGFR TKI as sequential therapy. The OS of these 173 patients was significantly worse than those who received 3rd G EGFR TKI as first‐line therapy (median 2.47 months vs. not reached, *p* = 0.0042) (Figure 5B).

**TABLE 6:** Factors associated with overall survival (OS) in the patients who received 3rd G EGFR TKI as first‐line and sequential treatment for T790M.

Variables	Univariate		Multivariable model	
	HR [95% CI]	*p*	HR [95% CI]	*p*
First‐line EGFR TKI: (vs. 1st G TKI)				
2nd G TKI	0.78 [0.34–1.77]	0.550	0.89 [0.34–2.34]	0.807
3rd G TKI	1.43 [0.68–3.02]	0.346	1.95 [0.80–4.77]	0.141
Male (vs. female)	1.51 [0.77–2.96]	0.229	2.71 [1.05–7.02]	0.039
Age (≥ 65 vs. < 65)	1.34 [0.70–2.58]	0.377	0.97 [0.46–2.04]	0.936
ECOG PS (≥ 2 vs. ≤ 1)	2.45 [1.11–5.41]	0.027	1.83 [0.69–4.87]	0.228
Smoking status (vs. never smoker)				
Current smoker	1.34 [0.18–9.89]	0.774	0.37 [0.04–3.79]	0.399
Ex‐smoker	2.14 [0.75–6.10]	0.156	1.15 [0.28–4.62]	0.848
Mutation type: (vs. Exon 19 deletion)				
L858R	1.28 [0.65–2.51]	0.470	1.49 [0.58–3.79]	0.408
Brain metastasis (vs. no)	1.12 [0.57–2.19]	0.750	0.57 [0.23–1.44]	0.234
Lung metastasis (vs. no)	2.26 [1.12–4.57]	0.024	3.19 [1.38–7.41]	0.007
Pleural metastasis/effusion (vs. no)	0.74 [0.58–0.94]	0.013	0.88 [0.37–2.12]	0.781
Bone metastasis (vs. no)	1.12 [0.59–2.14]	0.733	0.84 [0.38–1.84]	0.659
Liver metastasis (vs. no)	2.52 [1.22–5.22]	0.012	3.75 [1.54–9.11]	0.004
Pericardial metastasis/effusion (vs. no)	1.29 [0.57–2.94]	0.540	1.95 [0.62–6.11]	0.251
Adrenal metastasis (vs. no)	3.84 [1.34–11.02]	0.012	5.20 [1.48–18.29]	0.010
Other metastasis (vs. no)	1.21 [0.37–3.97]	0.748	1.12 [0.26–4.81]	0.877

*Note*: Data are presented in hazard ratio (HR) [95% confidence interval].

Abbreviations: 1st G, first‐generation; 2nd G, second‐generation; 3rd G, third‐generation; ECOG, Eastern Cooperative Oncology Group; EGFR, epidermal growth factor receptor; PS, performance status; TKI, tyrosine kinase inhibitor.

## 4 Discussion

In this study, we provide real‐world data on the clinical outcomes of patients with NSCLC harboring EGFR mutations who were treated with different generations of EGFR TKIs as their first‐line therapy against their disease. We also compared the clinical outcomes of patients receiving 3rd G EGFR TKI as first‐line therapy and as sequential therapy for T790M mutation. Our results showed that both PFS and OS were significantly different between those who received different generations of EGFR TKIs as first‐line therapy. Similar results were noted in the subgroup of patients with exon 19 deletion. In addition, 3rd G EGFR TKI provided the best PFS, particularly in the subgroup of patients with exon 19 deletion. In the subgroup of patients with exon 21 L858R mutation, those who received different generations of EGFR TKIs had similar PFS and OS. Among the patients who received re‐biopsy after 1st G or 2nd G EGFR TKI therapy, the T790M mutation detection rate was around 50%. The patients who received 1st G EGFR TKI with exon 19 deletion and a PFS ≥ 270 days had a significantly higher T790M mutation rate. The OS of the patients who received the 3rd G EGFR TKI as sequential therapy was similar to those who received 3rd G EGFR TKI as first‐line therapy. However, the OS of the patients who did not receive 3rd G EGFR TKI as sequential therapy was significantly worse than those who received 3rd G EGFR TKI as first‐line therapy.

Several phase III randomized controlled trials reported a median PFS of approximately 9–13 months in patients with advanced lung adenocarcinoma treated with 1st G or 2nd G EGFR TKIs as first‐line therapy [6, 7, 8, 9, 10]. The FLAURA trial reported that the patients who received osimertinib were associated with a longer PFS (median 18.9 vs. 10.2 months, *p* < 0.001) and OS (median 38.6 vs. 31.8 months, *p* = 0.046) than those who received 1st G EGFR TKIs. However, the OS of the patients with L858R who received osimertinib compared to those who received 1st G EGFR TKI was similar [18, 19]. In a retrospective cohort study of advanced EGFR‐mutant NSCLC patients, first‐line osimertinib showed similar OS to earlier‐generation EGFR TKIs (HR = 1.005, *p* = 0.969) [26]. In another retrospective study of NSCLC patients harboring exon 19 deletion or L858R mutation primarily treated with available EGFR TKIs, no difference in survival was observed between various TKIs. Benefit with osimertinib was observed only in the subgroup of patients with exon 19 deletion and baseline brain metastasis [27]. In addition, a real‐world study comparing the clinical outcomes between osimertinib and afatinib in advanced and recurrent NSCLC patients harboring EGFR mutations in Taiwan revealed no significant difference in PFS (median 18.8 vs. 13.1 months, *p* = 0.208) or OS (median not reached vs. 41.7 months, *p* = 0.552) (osimertinib vs. afatinib). Multivariable analysis in this study revealed that stage IVB disease and L858R were independent prognostic factors of poorer PFS. Stage IVB disease was the only prognostic factor of poorer OS [28]. We conducted the study to evaluate the clinical outcomes of different generations of EGFR TKIs as first‐line therapy. In our study, the PFS and OS were significantly different between different generations of EGFR TKIs as first‐line therapy. The patients who received 3rd G EGFR TKI as first‐line therapy had better PFS, particularly in the subgroup of patients with exon 19 deletion. In the subgroup of the patients with L858R, the OS of patients receiving 3rd G EGFR TKI as first‐line therapy was inferior to those receiving 2nd G EGFR TKI. The number of patients with L858R receiving 3rd G EGFR TKI in our study was relatively small, only nine patients. Besides, the patients receiving 3rd G EGFR TKI had a higher percentage of patients with independent prognostic factors of poorer OS (ECOG PS ≥ 2, 44% vs. 12%, and liver metastasis, 22% vs. 10%) than the patients receiving 2nd G EGFR TKI. Subsequent therapy was less frequent in the subgroup of patients receiving 1st G EGFR TKIs. In the patients receiving 1st G EGFR TKIs, the percentage of age older than 65 years (86%, *p* < 0.001) was higher, and that may be the reason that caused the less frequency of subsequent therapy in the subgroup of patients receiving 1st G EGFR TKIs.

T790M mutation is the most common on‐target mutation leading to drug resistance in EGFR‐TKI‐treated NSCLC patients, and it may be a favorable prognostic factor for both OS and PFS. Patients with lung adenocarcinoma and acquired T790M mutation who received osimertinib have been reported to have a more favorable outcome than those treated with standard platinum‐based chemotherapy [29]. The T790M mutation rate after disease progression with a first‐line EGFR TKI is approximately 50% [13, 14]. In an observational study of acquired EGFR T790M‐dependent resistance to EGFR TKI treatment in lung adenocarcinoma patients in Taiwan, the T790M acquisition rate was 52.8%. Patients with common baseline EGFR mutations, gefitinib (compared to erlotinib) administration, and longer treatment duration with EGFR TKIs had higher T790M occurrence [30]. The TERRA study, a retrospective, multicenter study conducted in Taiwan which evaluated the T790M detection rate after first‐line combination therapy with bevacizumab and an EGFR‐TKI in patients with advanced NSCLC, reported a T790M mutation rate after acquired resistance of 55.1%. Patients harboring exon 19 deletions had a relatively higher T790M mutation rate than those with exon 21 L858R mutation. The T790M mutation rate was significantly higher in patients who had PFS more than 12 months with first‐line combination therapy than in those who had PFS less than 12 months [31]. Another study showed that patients receiving 1st line afatinib had significantly lower T790M mutation rate than patients receiving 1st line gefitinib or erlotinib [32]. In our study, among the patients who received re‐biopsy after failure of frontline 1st/2nd G EGFR TKIs, the T790M mutation occurrence rate was 50%. The patients who received 1st G EGFR TKIs, with exon 19 deletion and PFS ≥ 270 days had a significantly higher T790M mutation rate.

Sequential therapy with osimertinib for T790M is the standard treatment subsequent to gefitinib, erlotinib, and afatinib. In the APPLE trial, a randomized, open‐label, noncomparative, multicenter, phase II study in treatment‐naïve patients with common sensitizing EGFR‐mutant advanced NSCLC, the patients receiving osimertinib as first‐line therapy had similar OS to those receiving sequential therapy (median NR vs. 84.4 months, HR, 1.01[90% CI, 0.61 to 1.68]) [33]. In our study, the OS of patients who received 3rd G EGFR TKI as first‐line therapy was not inferior to those who received 3rd G EGFR TKI as sequential therapy. However, not all of the patients who received 1st G or 2nd G EGFR TKIs had a drug resistance pathway involving T790M. In a previous study that evaluated the treatment outcomes of patients with EGFR‐mutant NSCLC who progressed on 1st G or 2nd G EGFR TKI therapy, the median OS of patients who developed the T790M mutation and had a history of osimertinib use was significantly longer than in patients with the T790M mutation but no history of osimertinib and in those who were T790M‐negative (57.0 vs. 32.9 vs. 39.0 months, *p* < 0.0001) [34]. In our study, the OS of patients who did not receive 3rd G EGFR TKI as sequential therapy was significantly worse than in those who received 3rd G EGFR TKI as first‐line therapy.

There are some limitations in this study. First, the number of patients who received 3rd G EGFR TKI was smaller compared to the number who received other EGFR TKIs. This was due to the regulations of the National Health Insurance (NHI) system in Taiwan. Gefitinib, erlotinib, and afatinib could be used as first‐line therapy for patients with advanced lung adenocarcinoma harboring sensitizing mutations during the study period, irrespective of brain metastasis status. However, osimertinib could be used as first‐line therapy only for exon 19 deletion‐positive, advanced lung adenocarcinoma patients without initial presentation of brain metastasis during the study period. This might explain the small number of patients treated with osimertinib, and also the uneven distribution of mutations in this group of patients in this study. Second, the choice of EGFR TKI by patients might be influenced by the clinician's personal preference. In our study, more patients received erlotinib. Third, only 57% of the patients were tested for T790M after disease progression, as the feasibility of re‐biopsy depended on the location of progression sites and the patients' clinical condition. Fourth, despite involving three different institutes, the sample size remains limited, which may affect the generalizability of our findings. Future research, including additional centers and larger populations, should be considered to validate and extend the results of our study. Finally, we did not analyze the adverse events between different generations of EGFR‐TKIs, leaving an important aspect of treatment outcomes unexplored.

In conclusion, our study demonstrated that patients with EGFR‐mutant NSCLC who received 3rd G EGFR TKI as first‐line therapy had the best PFS, especially the patients with exon 19 deletion. The OS of patients who received 3rd G EGFR TKI as first‐line therapy was not inferior to those who received 3rd G EGFR TKI as sequential therapy. However, not all patients who received 1st G or 2nd G EGFR TKIs were found to have drug resistance related to the T790M mutation. The OS of the patients who did not receive 3rd G EGFR TKI as sequential therapy was significantly worse than those who received 3rd G EGFR TKI as first‐line therapy. Based on our study, 3rd G EGFR TKI may provide better survival benefits as first‐line therapy for patients harboring sensitizing EGFR mutations, particularly those with exon 19 deletion.

## Conflicts of Interest

The authors declare no conflicts of interest.

## Data Availability

Data sharing not applicable to this article as no datasets were generated or analysed during the current study.
